# Fraudulent Medical Advertising

**Published:** 1888-03

**Authors:** 


### FRAUDULENT MEDICAL ADVERTISING.

A recent decision rendered in one of the courts in this State, in which the *State Board of Health*, which is charged with execution of the law governing the practice of medicine in the State of Illinois, is concerned, is of more than passing interest to the people of the State.

Under the above law, passed in 1877 by the Legislature, it is claimed that the State Board of Health has been enabled to rid the State of more than 3,000 unqualified practitioners of all kinds, and, what is of quite as much importance, has prevented many others from locating within its limits. Whilst this purification has been accomplished chiefly through the instrumentality of the medical profession, it is the general public that has been most benefited by the change, as was contemplated when the enactment of the law was urged in the interest of the citizens of the State. With commendable fidelity has the State Board of Health discharged the unacceptable duty devolved upon it by the law, and in so wise a manner has it acted as to have led to its imitation by boards organized in other States. Whilst thus struggling against prejudice and opposition, prompted by mercenary motives as manifested in many ways, it has encountered obstacles from a most unexpected source. The law provides that in case of abuse of the license to practice medicine issued by the Board of Health in accordance with its provisions, that board shall have power to revoke the license of such offender. Acting under the authority thus legally conferred upon it the board revoked, for alleged fraudulent medical advertising, the license of one of the most persistent of the medical peripatetics, and drove him from the State. Subsequently he returned, after having exhausted, as is supposed, other fields of his exploits, or been driven therefrom by medical-practice acts in force in them, and he now defies the authority of the board which revoked his license "for unprofessional and dishonorable conduct." In his efforts to prevent the Board of Health from driving him from the State again, he appealed to a court of law, and, in the decision above referred to, the action of the board in revoking his license was declared to be illegal, on the ground, as it is alleged, that the itinerant was not present to make a defense at the time of the revocation of his license. Since it appears to have been shown at the trial that the Board of Health made repeated, but ineffectual, efforts by legal notices, officially served on the offender, to secure his attendance at the meetings of the board when he was informed that his case was to be heard, and he persistently refused to be present at those meetings to make defense of his course, and the board only revoked his license after, seemingly, the most conclusive evidence of the correctness of the charges made against him, it is not clear upon what grounds it was decided that the course of the board had been illegal in revoking the license. If it be the ruling of courts that the board cannot put in execution the provisions of the medical-practice act in the absence of a culprit who refuses to present himself and defend his course, such ruling practically declares the law to be a nullity, and makes the way clear for a new influx of the unqualified and the unscrupulous.

